# Artificial Intelligence for Detection of Prognostically Significant Left Ventricular Dysfunction From Echocardiography

**DOI:** 10.1016/j.jacadv.2025.101891

**Published:** 2025-06-23

**Authors:** David Playford, Simon Stewart, Andrew Watts, Dean Kezurer, Yih-Kai Chan, Geoff Strange

**Affiliations:** a Institute for Health Research, The University of Notre Dame Australia, Fremantle, Western Australia, Australia; b BHF Cardiovascular Research Centre, University of Glasgow, Glasgow, United Kingdom; c Echo IQ Ltd, Sydney, New South Wales, Australia; d Mary MacKillop Institute for Health Research, Australian Catholic University, Melbourne, Victoria, Australia; e Heart Research Institute, University of Sydney, Sydney, New South Wales, Australia

**Keywords:** artificial intelligence, diastolic dysfunction, echocardiography, heart failure, left ventricular dysfunction, systolic dysfunction

## Abstract

**Background:**

Identification of left ventricular (LV) dysfunction following echocardiographic investigations remains problematic, particularly when the ejection fraction (EF) is preserved.

**Objectives:**

The authors examined the operational characteristics of artificial intelligence LV dysfunction (AI-LVD) identification from routinely obtained echocardiographic measurements.

**Methods:**

Following initial training in 126,136 (imputation cohort) and 254,735 (training cohort) cases from the National Echo Database of Australia, the AI-LVD was tested in 81,509 cases (last echo January 1, 2000-May 21, 2019) with no mitral valve intervention or pacemaker. This cohort comprised 41,796 men (51.3%) aged 62.3 ± 17.1 years and 39,713 women aged 63.2 ± 18.4 years, in whom 4,490 (5.5%), 3,734 (4.6%), and 59,297 (72.7%) had reduced, mildly reduced, and preserved EF, while 13,988 (17.2%) had no recorded EF and 39,940 (45.2%) had “indeterminate” filling pressures.

**Results:**

Overall, the AI-LVD generated a (sex-specific) output in decile distributions consistent with increasingly higher levels of LV dysfunction and mortality—actual 5-year mortality rising from 5.7% to 66.3% and 2.3% to 64.2% in men and women, respectively. The prognostic capacity of the AI-LVD persisted in preserved EF, when adjusting for age, year of echo, and missing echo parameters—with adjusted hazard for all-cause mortality during 1,541 (812-2,682) days follow-up 4.93-fold (95% CI: 4.35-5.59) and 7.11-fold (95% CI: 5.85-8.64) higher in the highest vs lowest decile group in men and women, respectively.

**Conclusions:**

A new AI-LVD algorithm using only echocardiographic measurements can reliably identify prognostically important LV dysfunction, including in preserved EF, even when key reporting parameters are missing. The AI-LVD can be used in real-time during routine echocardiography reporting.

Current American College of Cardiology, American Heart Association, and Heart Failure Society of America guidelines, along with the European Society of Cardiology heart failure (HF) guidelines, provide clinicians with clear left ventricular ejection fraction (LVEF) cutoffs to identify prognostically important levels of left ventricular systolic dysfunction (SD).^1^^,^^2^ Accordingly, many treatment decisions are made based on whether an individual's LVEF (commonly measured by transthoracic echo) is reduced (heart failure with reduced ejection fraction [HFrEF], <40%), mildly reduced (heart failure with mildly reduced ejection fraction [HFmrEF], 40 to <50%), or preserved (heart failure with preserved ejection fraction [HFpEF], ≥50%). Recent guidelines^3^ reflect the wealth of data in favor of medical therapy in patients with each form of HF,^4^ including sodium-glucose cotransporter type 2 inhibitor and glucagon-like peptide receptor agonists agonist trials in HFpEF.^5^, ^6^, ^7^ Despite these advances, there is less definitive evidence supporting treatment in the large majority of individuals with preserved LVEF,^8^ due in part to ongoing debate on the echocardiographic characteristics of HFpEF. Furthermore, since asymptomatic LV dysfunction falls within a spectrum that may lead to clinical HF, the American Heart Association/American College of Cardiology/Heart Failure Society of America guidelines classify 4 HF stages, including those at risk of HF (Stage A), LV dysfunction without clinical signs/symptoms of HF (Stage B), and symptomatic HF with evidence of congestion and/or elevations in biomarkers (Stages C and D).^2^ Recent reports from the National Echo Database of Australia (NEDA)^9^^,^^10^ have highlighted that LV dysfunction is heterogeneous, showing low long-term mortality when diastolic function is normal but progressively more adverse outcomes when diastolic function becomes more abnormal and/or LVEF decreases. In addition, there are important sex differences in the level of treatment response to HF therapies.^11^

Despite the best efforts of the American Society of Echocardiography (ASE) and other professional organizations to clarify how best to identify diastolic dysfunction (DD) in the setting of preserved EF and increased filling pressure in the setting of impaired EF, algorithm-based assessments remain challenging and underutilized. Moreover, in routine clinical practice, many of these patients are reported to have “indeterminate” diastolic function^11^—an important clinical issue that NEDA has previously confirmed, with 22% and 62% of those with LVEF >50% and ≤50%, respectively, falling into this category.^9^ Thus, there is substantial scope to facilitate more rapid and accurate detection of prognostically significant systolic and diastolic dysfunction in many people undergoing echocardiographic investigation.

Within the context of this common clinical uncertainty, we examined the operational characteristics and clinical potential of a novel LV dysfunction artificial intelligence (AI-LVD) algorithm trained to identify the phenotypical characteristics of LV dysfunction from routinely reported echo parameters including 4 specific groups: HFrEF, HFmrEF, preserved EF, and those cases routinely labeled “indeterminate/unknown” SD or DD. Specifically, we sought to determine if the probability output generated by the AI-LVD reliably identified increasing LV dysfunction and predicted a worse mortality outcome.

## Methods

### Study design

This was a retrospective, clinical cohort study based on the NEDA database,^12^ with model training and subsequent input data using only echocardiographic reported data (blinded to patient outcomes). In brief, NEDA collates and synthesizes routine echo reporting data from a large network of echocardiography clinics Australia-wide, with individual linkage to mortality outcomes. Consistent with Australia's subsidized health care system, echocardiography data are derived from a representative cohort of adults typically referred to investigate known or suspected heart disease. NEDA is registered with the Australian New Zealand Clinical Trials Registry (ACTRN12617001387314). Ethical approval has been obtained from all relevant Human Research Ethics Committees and the study adheres to the Declaration of Helsinki. Where appropriate, this study conforms to the “Standards for Reporting Diagnostic Accuracy Studies” guidelines.^13^

### AI-LVD development, training, and testing

**Figure 1:**
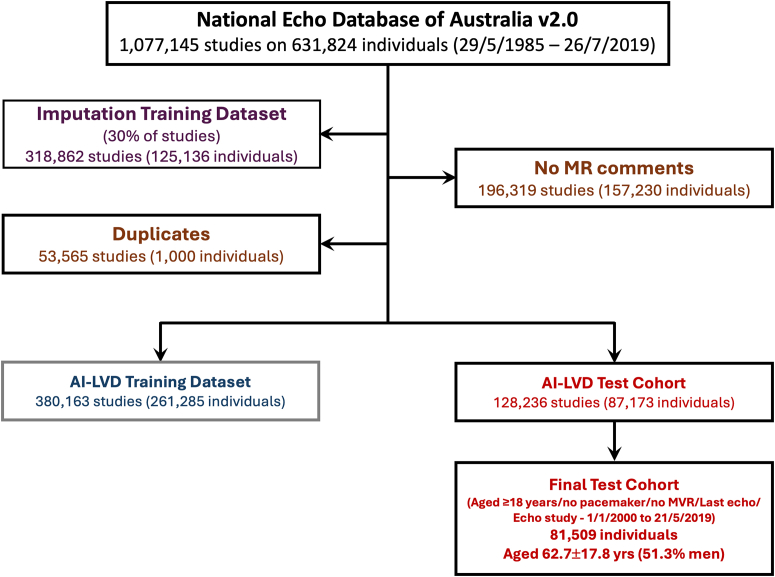
Study Flowchart This graph shows the distribution and flow of NEDA studies/reports and individual cases used to develop and test the AI-LVD (with key steps described in more detail in the Supplemental Material). Initially a random 30% split of the data set enabled training of an imputation model that accounted for missing measurement data. The remaining 70% of the data set underwent deployment of a natural language processing (NLP) AI system, followed by removal of echo studies with no valid comments on the echo report relating to the presence or absence of mitral regurgitation, and removal of studies where an individual with multiple studies was represented more than once in different data sets. The AI-LVD development process involved a random 75:25 split of the remaining database, with 75% to train the AI-LVD (after removal of repeat echo studies and prior mitral valve intervention). The final 25% was used to test the performance of the AI-LVD model after selecting the last echo for each patient, the absence of prior mitral valve intervention, age >18 years, and echo between January 1, 2000, and the census date. AI-LVD = artificial intelligence-based detection of left ventricular dysfunction; MR = mitral regurgitation; MVR = mitral valve replacement; NEDA = National Echo Database Australia.

We have previously reported the successful development of a stand-alone AI algorithm to reliably detect severe forms of aortic stenosis from routine echo measurement data.^14^^,^^15^ Early in the AI-LVD development, we intended to create a similar stand-alone AI algorithm to automatically predict mitral regurgitation (MR) severity. However, while this proved challenging, serendipitously, the AI outputs from the initial “training cohort” (see Figure 1 and below) demonstrated its capacity to detect sets of echocardiographic measurements associated with worsening LV dysfunction characteristically associated with the clinical syndrome of HF^16^ and a poor prognosis,^9^^,^^10^ and in many more cases than can be identified from traditional guideline application. We therefore focused on the AI's capacity to detect LV dysfunction by more closely examining its operational characteristics in a new “test” data set, not previously analyzed by the AI.

The AI-LVD algorithm development involved a binary classifier due to its superior performance compared with other computational methods explored during development and an AutoPyTorch package. Figure 1 summarizes the steps taken to train and test the model. In summary, the NEDA data set (v2.0) underwent proprietary (Echo IQ Ltd) AI imputation modeling to account for missing data (excluding data used to train the imputation model), after which studies containing no valid comments indicating presence or absence of MR (the initial AI training model) and mitral valve repair or replacement were excluded along with 53,565 studies where an individual was found more than once in different data sets (due to having multiple studies performed). This process ensured that every individual was encountered only once across data sets and had not been involved in prior model training. The remaining 508,399 studies were then randomly split into 380,163 studies from 261,285 people (75%) to train the AI-LVD, and 128,236 studies from 87,173 people (25%) for model testing. The final test cohort (the focus of this report) comprised 81,509 individuals selected for their last recorded echo, aged ≥18 years and with no reports of mitral valve repair, replacement, or pacemaker. The final AI-LVD model has been made available for research purposes through the corresponding author, and the PRIME checklist^17^ summary table is shown in Supplemental Table 2.

### Echocardiographic data

NEDA captures real-world echocardiographic-derived numerical data topped-up with natural language processing extraction of key text reports. As is the case with review by a physician, the model underpinning the development of the finalized AI-LVD relies on all available data. A complete set of measurements is generated by the previously described imputation model.^14^ Briefly, the model ingests up to 119 (numerical structured report) echo measurements from the exam (without reference to the cardiologist/physician comments within the report) and automatically fills in missing data. The model shows excellent capacity to predict missing data.^14^ The complete set of echo measurements is then presented to the AI-LVD model. Using Python 3.8.10, the publicly available automated machine learning software AutoPyTorch (version 0.2.1) was used to train an ensemble classifier to predict moderate or greater MR. The software generates and trains a large number (n = 50) of neural networks and traditional machine learning algorithms to predict the target label. Bayesian optimization is used to select a weighted ensemble of the models to produce a final classifier. During AI-LVD model development, age and sex were added as nonimputed inputs to the trained AI model and highly correlated columns were removed from the imputation model outputs in order to improve model performance and explainability, for a total of 49 input measurement features. Detailed explainability experimentation was then performed (informed by consultation with a panel of experts, Supplemental Material), first confirming the AI-LVD's capacity to detect increasing levels of LV dysfunction, followed by feature importance of the AI model using Individual Conditional Expectation graphs and Partial Dependence Plots^18^ in both 1D and 2D (Supplemental Figures 1 and 5). Further experimentation starting with zero inputs and increasing the number of key variables as inputs showed the model's increasing performance with higher numbers of key variables (Supplemental Figure 2). The model was intentionally left uncalibrated, reflecting future intended clinical use of the product.

Considering the proportion of echo reports in which these specific variables were documented, and by removing all other columns to simulate a clinical environment in which only a limited number of variables could be collected, it was shown that 8 echo variables were sufficient to maintain a high fill rate and high fidelity in the identification of patients at risk of clinically significant LV dysfunction. This enabled an accurate picture of the AI-LVD's key features of importance in the output to be identified. In addition to the mandatory consideration of the age and sex of each person, these key features were body surface area, left atrial volume index (LAVi), two-dimensional left ventricular septal and posterior wall thickness, left ventricular diastolic dimension (thereby enabling LV mass index to be calculated according to the ASE method^19^), and LV systolic dimension (enabling Teichholz LVEF measurement and ventricular volumes). Supplemental Figure 3 shows the proportion of echo reports in which these specific variables were documented (and therefore nonimputed by the AI) on a sex-specific basis, within the final test cohort of 81,509 cases.

### Classification of LV systolic and diastolic dysfunction

For all the primary analyses, where the LVEF was documented (67,521/81,509 cases), independent of/blinded to AI-LVD outputs, we categorized cases as having HFrEF (LVEF ≤40%), HFmrEF (LVEF 40% to 49.9%), preserved LVEF (LVEF ≥50%), or “unknown” systolic function using the standard LVEF cutoffs recommended in the most recent European Society of Cardiology HF guidelines. Consistent with a previous analysis of the NEDA cohort, we also applied ASE/European Association for Cardiovascular Imaging (EACVI) guideline-derived categories of DD. Accordingly, for those categorized with preserved LVEF, we used the 4 recommended parameters of DD (E:e' ratio, septal/lateral e' velocity, tricuspid regurgitation (TR) peak velocity, and LAVi) to further categorize cases with “*normal*” (<50% of parameters abnormal), “*indeterminate*” (50% of parameters abnormal), and “*abnormal*” (>50% parameters abnormal) diastolic function. Likewise, specific guideline criteria were applied to the HFrEF and HFmrEF categories using the recommended parameters (E:A ratio, and where the E:A and E velocity were indeterminate, the E:e' ratio, TR peak velocity, and LAVi) to derive the output categories of “*normal*” (grade I DD), “*indeterminate*,” and “*increased*” filling pressure (combining grades II and III DD^20^).

### Study outcomes

The primary study outcome was the operational characteristics of the AI-LVD probability output (on a sex-specific basis) in: 1) correlating with a pattern of increasing LV dysfunction (where echo data were available to confirm this); and 2) discriminating between those with an adverse prognosis, regardless of whether the traditional measures of SD and DD were reported. Prospectively, we applied the statistical (in decile groups) distribution of AI-LVD probability output to examine these. Actual 1- and 5-year follow-up (based on a minimum of 365 days and 1,825 days follow-up before census, respectively) was calculable in 71,157 (97.1%) and 45,510 (55.8%) of cases. Overall, there were 21,306 all-cause deaths (26.1%), comprising 11,554/41,796 (27.6%) male and 9,752/39,713 (24.6%) female deaths, during a median follow-up of 1,541 (IQR, Q1-Q3: 812-2,682) days.

### Statistical analyses

Given the cohort size and deaths available for analysis, no formal power calculations were conducted. Standard methods for describing and comparing grouped data, including mean ± SD, median (IQR), and proportions according to baseline profiling (last echocardiogram) were applied. Sex-specific Cox proportional hazard models (entry model with proportional hazards confirmed by visual inspection) adjusted for age, year of (last) echo (reference group for comparison–2000-04), the number of key echo variables reported/available to the AI-LVD (0-8), and the decile distribution of AI-LVD output (reference group for comparison–lowest decile group being an output probability output of <0.422) were used to generate HRs with 95% CIs for all-cause mortality (with censored events) during entire follow-up. All statistical analyses were performed using SPSS (Statistical Package for the Social Sciences) version 29.0 software (SPSS Inc). Significance was accepted at the level of *P* < 0.05 (2-sided).

## Results

### Study cohort

Table 1 summarizes the profile of the test cohort of 81,509 individuals at last echo according to their biological sex and EF category. Overall, the cohort comprised 41,796 men (51.3%) aged 62.3 ± 17.1 years and 39,713 women (48.7%) aged 63.2 ± 18.4 years. Based on their documented LVEF, 4,490 (5.5%), 3,734 (4.6%), and 59,297 (72.7%) of all cases were categorized as having HFrEF, HFmrEF, and preserved EF, respectively, with the remaining 13,988/81,509 cases (17.2%) remaining unclassified. Applying guideline criteria, 6.5% and 15.4% of men and 7.7% and 16.9% of women, respectively, had definitive evidence of abnormal DD and/or elevated filling pressures. However, 44.5% of men and 46.2% women had “indeterminate” filling pressures—the majority of these (when LVEF was documented) with a preserved EF (60.8%).

**Table 1:** Cohort Characteristics for Men and Women, and According to Left Ventricular Systolic Function

	Sex-Specific Profile		Profile According to Left Ventricular Systolic Function			
	Men (n = 41,796)	Women (n = 39,713)	Reduced EF (n = 4,490)	Mildly Reduced EF (n = 3,734)	Preserved EF (n = 59,297)	Rest (EF Unknown) (n = 13,988)
Baseline						
Age (last echo), y	62.3 ± 17.1	63.2 ± 18.4	57.4 ± 17.4	65.5 ± 14.3	69.7 ± 13.5	70.2 ± 12.7
Women, %	-	-	1,337 (29.8%)	1,278 (34.2%)	30,650 (51.7%)	6,448 (46.1%)
Body mass index, kg/m^2^	28.1 ± 5.56	27.9 ± 6.95	27.5 ± 6.28	28.1 ± 6.29	28.0 ± 6.23	28.4 ± 6.75
Systolic/Diastolic BP, mm/Hg	134 ± 20.8/ 76.9 ± 11.1	136 ± 23.4/ 77.0 ± 11.3	126 ± 23.2/ 73.0 ± 13.2	131 ± 21.8/ 75.0 ± 10.7	136 ± 21.8/ 77.4 ± 10.9	125 ± 25.1/ 71.5 ± 13.6
Right heart dimensions/function						
TR peak velocity, m/s	2.61 ± 0.47	2.60 ± 0.48	2.85 ± 0.52	2.71 ± 0.51	2.57 ± 0.46	2.72 ± 0.54
RA volume index, mL/m^2^	39.6 ± 27.3	34.8 ± 25.1	53.2 ± 33.0	40.5 ± 25.0	36.5 ± 25.9	35.3 ± 19.6
Left heart dimensions/function						
AV peak velocity, cm/s	1.56 ± 0.65	1.59 ± 0.59	1.54 ± 0.76	1.54 ± 0.68	1.57 ± 0.60	1.59 ± 0.67
LAVi, mL/m^2^	46.0 ± 31.4	42.3 ± 27.8	61.9 ± 40.0	48.9 ± 28.3	65.2 ± 8.03	-
LVEF, %	59.6 ± 13.0	64.1 ± 11.1	30.5 ± 8.00	45.4 ± 2.59	65.2 ± 8.02	-
Stroke volume index, mL/m^2^	40.7 ± 13.3	39.6 ± 12.1	32.4 ± 11.5	37.6 ± 10.8	41.2 ± 12.8	35.3 ± 19.6
Mitral E/e' ratio	10.5 ± 4.79	11.0 ± 5.18	8.06 ± 2.12	8.49 ± 2.02	8.81 ± 2.17	8.71 ± 2.35
Mitral E velocity, cm/s	77.4 ± 24.1	83.3 ± 25.8	84.9 ± 29.7	79.1 ± 29.3	80.2 ± 24.3	79.4 ± 26.9
LV mass index (ASE), g/m^2^	100.0 ± 30.4	86.3 ± 28.1	125.6 ± 37.0	108.6 ± 32.2	90.9 ± 28.1	86.7 ± 31.9
Normal diastolic function	23,992 (57.4%)	24,259 (61.1%)	-	-	33,096 (55.8%)	13,998 (100.0%)
Abnormal diastolic function	2,727 (6.5%)	3,075 (7.7%)	4,490 (100%)	3,734 (100%)	4,672 (7.9%)	-
Normal filling pressures %	5,883 (14.1%)	6,853 (17.3%)	-	-	12,736 (21.5%)	-
Indetermined filling pressure, %	18,579 (44.5%)	18,361 (46.2%)	-	910 (24.4%)	36,030 (60.8%)	-
Elevated filling pressure, %	6,641 (15.4%)	6,714 (16.9%)	4,490 (100%)	2,824 (75.6%)	10,531 (17.8%)	-
AI-LVD profile						
Key echo variables reported, 0-8	4.57 ± 2.22	4.36 ± 2.21	4.51 ± 1.64	4.58 ± 1.63	5.22 ± 1.67	1.18 ± 1.37
AI-LVD probability output, 0-1.00	0.453 ± 0.037	0.458 ± 0.039	0.516 ± 0.040	0.482 ± 0.042	0.447 ± 0.032	0.468 ± 0.033
Follow up						
All-cause mortality, %	11,554 (27.6%)	9,752 (24.6%)	2,480 (55.2%)	1,480 (39.6%)	12,599 (21.2%)	4,747 (33.9%)
Actual 1-y mortality, %	4,320 (10.6%)	3,245 (8.4%)	1,215 (28.0%)	568 (15.8%)	3,696 (6.4%)	2,086 (15.0%)
Actual 5-y, mortality, %	6,329 (27.1%)	5,139 (23.2%)	1,542 (56.5%)	843 (41.3%)	6,660 (19.5%)	2,423 (37.2%)

Number of cases with a documented – body mass index (n = 59,332), systolic (n = 11,925)/diastolic (n = 11,876) blood pressure (BP), tricuspid regurgitation (TR) peak velocity (n = 44,772), right atrial (RA) volume index (n = 22,080), indexed left atrial volume (LAVi) (n = 28,386), left ventricular ejection fraction (LVEF) (n = 67,521), stroke volume index (n = 22,234), ratio of mitral E wave to myocardial Doppler septal e' velocity (E/e' ratio) (n = 36,842), E wave velocity (n = 65,203), and LV mass index calculated using the American Society of Echocardiography (ASE) method (n = 47,791). Full 1- and 5-year mortality calculable in 71,600 and 34,515 cases, respectively. Key echo variables are based on the 8 clinical variables identified as the most important in deriving the AI-LVD. The methodology and explainability is described in more detail in the text and Supplemental Material.

### AI-LVD probability output

Despite frequent missing data in this real-world echocardiography database (with mean availability of 4.6 ± 2.2 and 4.4 ± 2.2 of the 8 echo key variables present among men and women, respectively), the AI-LVD generated an output for every case with no “indeterminate” outputs, with a mean probability output of 0.45 ± 0.04 for men and 0.46 ± 0.04 for women. Supplemental Figure 2 shows that the (combined for men and women) distribution of AI-LVD output became progressively more representative as the number of key echo variables increased, with body surface area, LVEF, and E wave velocity, along with the age and sex of each case, being considered operationally critical. Supplemental Table 1 summarizes the echo characteristics of men and women combined, according to the decile distribution of AI-LVD output. Consistent with the observation during early development of the algorithm, each decile increment indicated increasing levels of SD and DD (where known/quantifiable).

### Actual 1- and 5-year mortality

The right panel of the Central Illustration shows the unadjusted pattern of actual 1- and 5-year mortality for men (top graph) and women (bottom graph) according to the sex-specific, decile distribution of AI-LVD output. In both sexes, there was a stepwise increase in mortality according to the decile distribution of the output, but with potentially important differences. Among men, 1-year mortality rose from 1.8% to 32.7% in the lowest to highest decile groups, rising to >10% within the 4th highest decile group. In women, the equivalent rates rose from 0.9% to 27.5%, with 1-year mortality rising >10% in the 3rd highest decile group. This association (of low to increasing mortality) was maintained when examining actual 5-year mortality, with the highest 2 decile groups in both sexes being associated with very poor survival (49% to 64%). While mortality rates steadily increased in men (from a low of 5.7% to 66.3%), there was a more noticeable increment of >8% mortality in women between the 5th and 6th deciles of AI-LVD (overall range 2.3% to 64.2%).

**Central Illustration:**
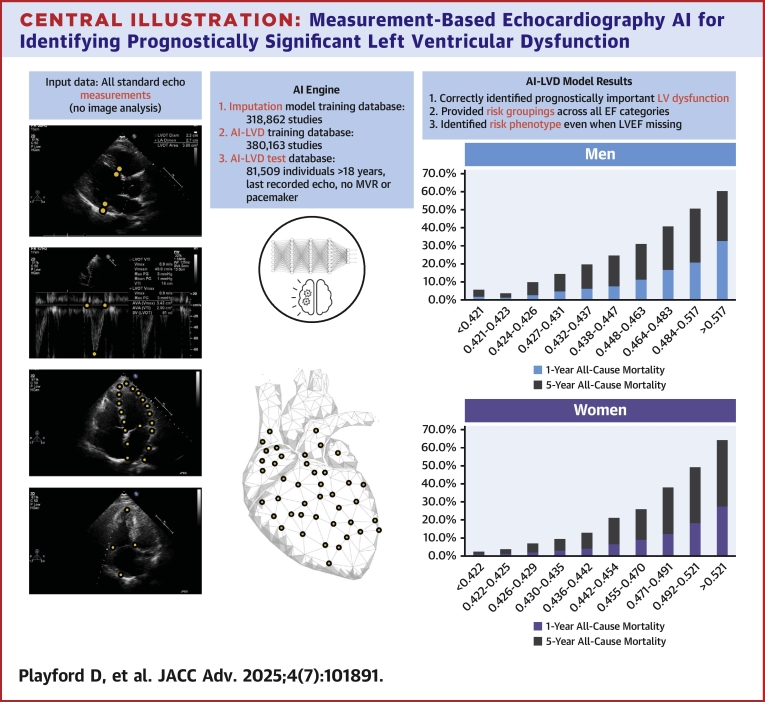
Measurement-Based Echocardiography AI for Identifying Prognostically Significant Left Ventricular Dysfunction Echocardiographic measurement data (no image analysis) indicated in the left panel is taken into the AI model (middle panel), producing a probability output. The right panel demonstrates the unadjusted pattern of actual 1- and 5-year mortality for men (top graph) and women (bottom graph) according to the sex-specific, decile distribution of AI output. LVEF = left ventricular ejection fraction; MVR = mitral valve replacement; other abbreviation as in Figure 1.

### Long-term all-cause mortality

**Figure 2:**
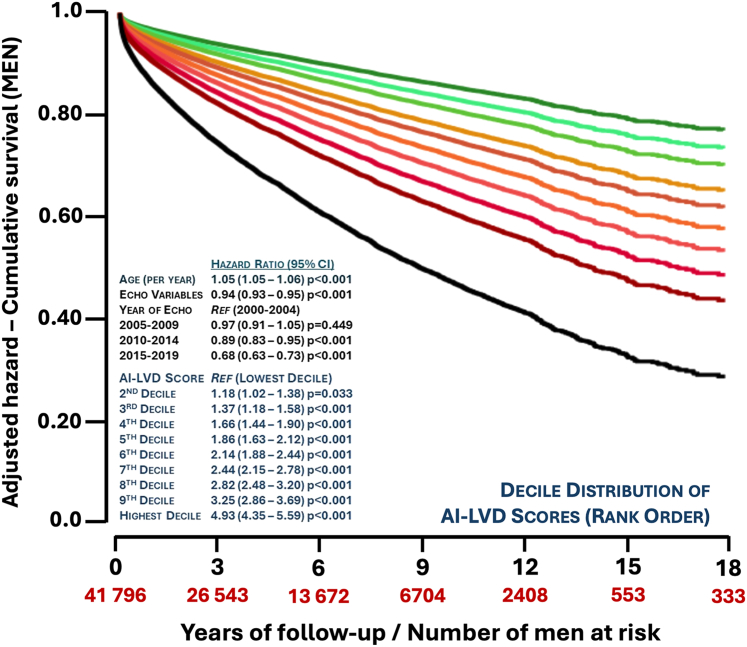
Adjusted Risk of Long-Term, All-Cause Mortality According to the AI-LVD Probability Output (Men) This graph plots the Cox proportional hazards for long-term all-cause mortality adjusted for age, year of (last) echo (reference group for comparison – 2000-04), the number of key echo variables reported/available to the AI-LVD (0-8), and the decile distribution of AI-LVD output in men (reference group for comparison – lowest decile group being an output probability of <0.421). Abbreviations as in Figure 1.

**Figure 3:**
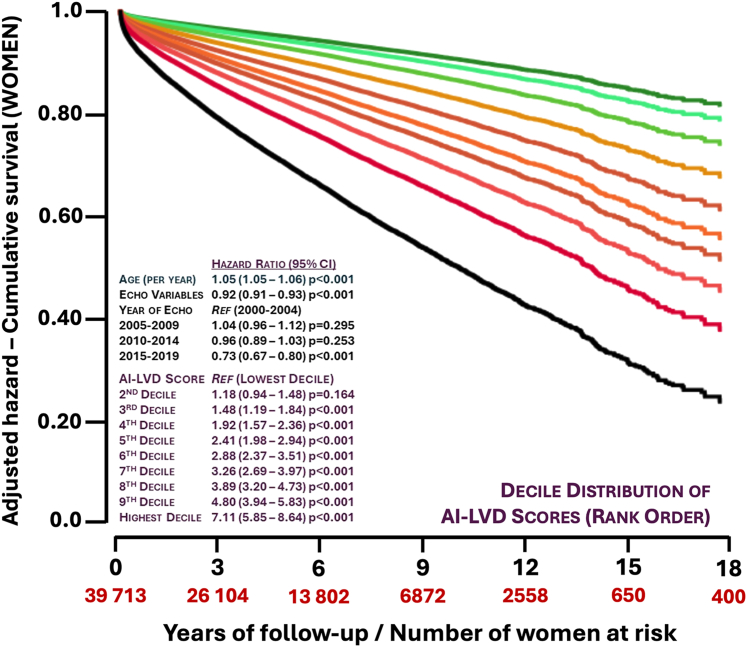
Adjusted Risk of Long-Term, All-Cause Mortality According to the AI-LVD Probability Output (Women) This graph plots the Cox proportional hazards for long-term all-cause mortality adjusted for age, year of (last) echo (reference group for comparison – 2000-04), the number of key echo variables reported/available to the AI-LVD (0-8), and the decile distribution of AI-LVD output in women (reference group for comparison – lowest decile group being an output probability output of <0.422). Abbreviations as in Figure 1.

When considering the entirety of follow-up, the same pattern of increasing mortality according to the sex-specific, decile distribution of AI-LVD output was evident, ranging from 7.2% to 64.8% in men and 3.0% to 63.0% in women. As shown in Figure 2 (men) and Figure 3 (women), when adjusting for age, the number of key echo variables used by the AI algorithm and year of echo, there was a clear gradient of increasing mortality associated with the decile distribution of the AI-LVD output that became more apparent over time. Of note in Figure 2, for men, the adjusted risk of mortality (compared to the reference group/lowest decile of AI-LVD output) rose above 1.5-fold by the 4th decile group (aHR: 1.66; 95% CI: 1.44-1.90), with the 10% of “highest” risk men having a close to 5-fold increased risk of dying (aHR: 4.93; 95% CI: 4.35-5.59). As also shown in Figure 3, a similar pattern of increasing mortality for women was observed, with a steeper gradient of an increasing risk evident according to the decile distribution of the AI-LVD output (the aHR rising from 1.92, 95% CI: 1.57-2.36 to 7.11, 95% CI: 5.85-8.64 from the 4th to highest decile groups). As shown in Supplemental Figure 4, a similar pattern was again observed in those with HFrEF, HFmrEF, preserved LVEF, and “indeterminate” cases.

## Discussion

Applying the big data approach of NEDA we demonstrate that a readily applicable AI-LVD algorithm (that may be deployed in real-time during echocardiography) can identify prognostically important LV dysfunction, and thereby has potential to assist in the diagnosis of HF. Overall, the distribution and prognostic power of the AI-LVD probability output generated from >81,000 people routinely investigated with echo, showed clear and consistent markers of worsening LV dysfunction within HFrEF, HFmrEF, and preserved EF categories. Consistent with the observed deteriorating pathophysiology, an increasing AI-LVD probability output showed increasing sex-specific actual 1-year, actual 5-year, and overall (censored) all-cause mortality up to 16-year follow-up across all subgroups. On an adjusted basis, this delineation in mortality persisted for both men and women. It is important to consider that the AI-LVD algorithm often operated efficiently under a “handicap” of limited information (ie, the minimum fundamental echo variables identified by expert clinicians as instrumental in assessing SD/DD). Clearly, this required higher levels of imputation to derive an output. Nevertheless, even in the real-world practice of incomplete reporting/documentation of echocardiographic measurement data, cogent outputs (in respect to mortality outcomes) were generated. This may offer a potential advantage over the current ASE/EACVI diastolic function algorithm, which cannot attribute a diastolic function assessment in 44.5% of men and 46.2% women (predominantly with a preserved EF), and highlights the need for simple automated systems that may assist clinician diagnosis.^21^

Given that the original intention was to develop an algorithm that could readily identify otherwise unreported MR, it is relevant to ask why the AI-LVD has proven more adept in detecting increasing levels of prognostically important LV dysfunction. The common pathology in both LV dysfunction and MR is left ventricular dilatation, increased left ventricular mass, abnormal myocardial relaxation velocities, increased left atrial volumes and filling pressure, development of pulmonary hypertension and eventual right-sided chamber dysfunction. In more simplistic terms, it appears that an echo training model based on the documented evidence of MR was more effective in detecting the characteristics associated with LV dysfunction rather than one specific condition. Reporting of HFrEF and HFmrEF relies on a single number (the LVEF), whereas diastolic function assessment requires several parameters that have significant variability,^22^ although recent use of AI image analysis improves reliability of these measures,^23^ including by nonexpert users.^24^ Additional measures such as global longitudinal strain and atrial strain provide confirmatory information^25^^,^^26^ and can be useful in predicting HF outcomes.^27^ In contrast to image-based AI systems, the current AI-LVD algorithm uses all available echocardiographic measurements, thereby showing potential to robustly identify LV dysfunction phenotypes. The AI-LVD's use of multiple echocardiographic variables has high relevance to the rapid detection of potential HF, whereby there are multiple pathways (including a diverse range from ischemic cardiomyopathy to hypertensive heart disease) to this increasingly common clinical syndrome.^28^ As shown by our findings, in the absence of any granular clinical data, the simple presence of AI-identified LV dysfunction conveys an increasing risk of mortality, irrespective of whether a clinical diagnosis of HF was made.

In explicitly acknowledging the AI-LVD algorithm has yet be tested and proven (in an independent and blinded fashion) outside of the NEDA cohort, we are not (yet) suggesting that it is ready to be applied on a routine clinical basis. We have initiated further studies assessing the AI-LVD algorithm's capacity to detect clinically adjudicated HF (including HFrEF, HFmrEF, and HFpEF), along with identification of patients who may develop future de novo HF if this diagnosis is not clinically apparent at baseline. If these studies demonstrate the AI-LVD's capacity to predict both LV dysfunction and HF, this technology has future potential to be used in parallel with echocardiographic reporting, and potentially augment clinical HF scores^29^^,^^30^ to assist the clinician with the goal of instituting appropriate medical therapy to improve outcomes.^31^^,^^32^

### Study Limitations

Despite the encouraging results using the AI-LVD algorithm, several limitations need to be highlighted. NEDA does not (yet) capture adjudicated HF diagnoses, and independent testing of the AI-LVD in a separate cohort of patients with and without clinically and biomarker-confirmed HF is needed. NEDA also does not (yet) capture each comorbidity that may result in LV dysfunction, or potentially contribute to mortality outcomes. Similarly, HF hospitalization events were not evaluated in this study. Although a detailed prosecution of the AI-LVD's capacity to detect individual underlying causes of LV dysfunction is required, the system overall has demonstrated excellent capacity to predict underlying risk. We cannot exclude the possibility that the AI is identifying additional elements that may have been misattributed to LV dysfunction. Moreover, some patients with clinical HF may be missed by the AI-LVD. However, each of the echocardiographic measurements known to be abnormal in HF featured as important variables (such as LVEF, LV dimensions, e' velocity, mitral E and A velocities, left atrial volumes, TR velocity, and right heart dimensions). We have not yet tested the capacity of the AI-LVD to predict outcomes compared with newer modalities such as LV or LA strain. We will actively address these shortcomings in our planned future studies in external data sets. Finally, although Australia is a multicultural nation representing many nationalities and different racial groups, we have not specifically tested the AI-LVD system in different ethnic groups.

## Conclusions

Applied to routinely acquired echocardiographic measurement data, including cases where a limited number of echo measurements were reported, we verified that an AI-LVD algorithm can reliably identify phenotypic abnormalities of LV dysfunction in patients at high risk of developing HF and, most critically, at risk of premature mortality. The AI-LVD output identified worsening LV dysfunction for each LVEF category (specifically, further risk stratifying beyond the single dimension of LVEF in HFrEF, HFmrEF, and preserved EF categories), and the associated increased short- and long-term mortality. The AI-LVD also offers a potential advantage over the current ASE/EACVI diastolic function algorithm, which cannot attribute a diastolic function assessment in close to half of assessments (predominantly with a preserved EF). Further clinical validation of the AI-LVD will involve application to external data sets with objectively adjudicated clinical HF diagnoses and related hospitalizations. This will help determine the potential clinical utility of the AI-LVD to identify prognostically relevant LV dysfunction, assist in clinical HF diagnosis, and predict future HF hospitalization events.

## Funding support and author disclosures

Dr Playford has received consulting fees from Echo IQ and investigator fees from NEDA. Dr Stewart is supported by the 10.13039/501100000925National Health and Medical Research Council of Australia (GNT1135894); has received consulting fees from Echo IQ and NEDA; and is the Director of Research for NEDA. Dr Watts is an employee of Echo IQ. Dr Strange has received consulting fees from Echo IQ and investigator fees from NEDA. All other authors have reported that they have no relationships relevant to the contents of this paper to disclose.

Perspectives **COMPETENCY IN PRACTICE-BASED LEARNING:** Accurate diagnosis of heart failure is an essential skill for physicians and echocardiography is a cornerstone of diagnosis. Interpretation of echocardiography requires subspecialty knowledge, and novel automatic approaches that assimilate echocardiographic data are needed to assist physician interpretation. We have developed a measurement-based AI system where the probability output identifies prognostically important left ventricular dysfunction, even when key parameters (such as ejection fraction) are missing.

**TRANSLATIONAL OUTLOOK:** This AI shows promise as a tool to assist physicians in the timely and accurate diagnosis in each LVEF category of heart failure. If used optimally during echocardiographic interpretation, this discovery has the potential to improve the precision of physician diagnosis and application of guideline-directed medical therapy.
