# Supplementary material for: Artificial Intelligence for Detection of Prognostically Significant Left Ventricular Dysfunction From Echocardiography
Source: JACC Adv. 2025 Jun 23;4(7):101891. doi: 10.1016/j.jacadv.2025.101891 (PMC12240068; doi:10.1016/j.jacadv.2025.101891)
Supplement: Supplementary data [file mmc1.docx]

**Supplemental Table 1: The echocardiographic characteristics of men and women combined, according to each increasing decile of LVD-AI probability output**

|  | **Decile 1**  (n=8061) | **Decile 2**  (n=8405) | | **Decile 3**  (n=8186) | | **Decile 4**  (n=8206) | | **Decile 5**  (n=8146) | | **Decile 6**  (n=7714) | | **Decile 7**  (n=7411) | **Decile 8**  (n=7510) | **Decile 9**  (n=8056) | **Decile 10**  (n=9814) |
| --- | --- | --- | --- | --- | --- | --- | --- | --- | --- | --- | --- | --- | --- | --- | --- |
| **Baseline** | | | | | | | | | | | | | | | |
| Age (last echo), years | 47.1±14.7 | 49.3 ± 15.7 | | 52.9 ± 16.4 | | 57.8 ± 15.8 | | 61.6 ± 15.4 | | 64.7 ± 14.8 | | 68.0 ± 14.1 | 72.1 ± 1.7 | 75.3 ± 11.7 | 77.4 ± 12.2 |
| Women, % | 3146 (39.0%) | 3897 (46.4%) | | 3898 (47.6%) | | 3966 (48.3%) | | 3879 (47.6%) | | 3958 (51.3%) | | 3787 (51.1%) | 3674 (48.9%) | 4162 (51.7%) | 5346 (54.5%) |
| Body mass index, kg/m^2^ | 28.0 ± 6.0 | 28.0 ± 6.2 | | 28.3 ± 6.4 | | 28.5 ± 6.3 | | 28.6 ± 6.5 | | 28.3 ± 6.4 | | 27.9 ± 6.3 | 27.8 ± 6.2 | 27.6 ± 6.2 | 26.5 ± 6.0 |
| Systolic/Diastolic BP, mm/Hg | 130 ± 17 /  78 ±10 | 130 ± 19 /  77 ± 11 | | 131 ± 20 /  78 ± 11 | | 134 ± 21 /  78 ± 11 | | 136 ± 21 /  78 ± 11 | | 137 ± 22 /  77 ± 11 | | 138 ± 22 /  77 ± 11 | 138 ± 22 /  76 ± 12 | 139 ± 25 /  76 ± 12 | 136 ± 25 /  75 ± 13 |
| **Right heart dimensions/function** | | | | | | | | | | | | | | | |
| TR peak velocity, m/s | 2.24 ± 0.27 | 2.27 ± 0.28 | | 2.34 ± 0.30 | | 2.42 ± 0.34 | | 2.51 ± 0.36 | | 2.58 ± 0.40 | | 2.65 ± 0.43 | 2.71 ± 0.44 | 2.80 ± 0.46 | 3.05 ± 0.50 |
| RA volume index, ml/m^2^ | 19.5 ± 6.4 | 23.5 ± 10.4 | | 28.4 ± 15.2 | | 33.9 ± 19.7 | | 41.3 ± 23.7 | | 44.7 ± 25.8 | | 48.2 ± 28.1 | 52.3 ± 30.8 | 56.7 ± 31.5 | 68.6 ± 34.8 |
| **Left heart dimensions/function** | | | | | | | | | | | | | | | |
| AV peak velocity, m/s | 1.35 ± 0.28 | 1.39 ± 0.33 | | 1.42 ± 0.37 | | 1.47 ± 0.44 | | 1.51 ± 0.50 | | 1.58 ± 0.57 | | 1.65 ± 0.67 | 1.75 ± 0.77 | 1.81 ± 0.82 | 1.84 ± 0.91 |
| LAVi, ml/m^2^ | 23.7 ± 7.2 | 28.4 ± 10.9 | | 33.6 ± 15.8 | | 39.3 ± 19.8 | | 46.0 ± 23.7 | | 49.4 ± 25.5 | | 53.2 ± 28.4 | 57.5 ± 30.4 | 62.7 ± 31.8 | 81.6 ± 45.5 |
| LVEF, % | 66.6 ± 6.4 | 65.1 ± 7.1 | | 64.8 ± 7.9 | | 64.5 ± 9.0 | | 64.0 ± 10.0 | | 63.0 ± 11.0 | | 62.0 ± 11.9 | 60.8 ± 12.6 | 57.7 ± 14.3 | 49.2 ± 17.8 |
| Stroke volume index, mL/m^2^ | 37.4 ± 8.6 | 38.4 ± 8.8 | | 39.4 ± 9.3 | | 40.6 ± 9.9 | | 41.6 ± 10.9 | | 41.9 ± 11.4 | | 42.7 ± 12.4 | 43.0 ± 12.9 | 41.5 ± 14.6 | 37.1 ± 19.7 |
| Mitral E/e' ratio | 7.4 ± 2.0 | 8.0 ± 2.2 | | 8.7 ± 2.4 | | 9.5 ± 2.8 | | 10.5 ± 3.1 | | 11.5 ± 3.3 | | 12.3 ± 3.8 | 13.7 ± 4.7 | 15.4 ± 5.6 | 18.8 ± 8.2 |
| Mitral E wave velocity, cm/s | 69.5 ± 15.9 | 72.8 ± 17.6 | | 74.4 ± 18.5 | | 74.0 ± 18.7 | | 74.3 ± 19.6 | | 76.2 ± 20.3 | | 79.5 ± 21.7 | 84.6 ± 23.1 | 94.0 ± 25.7 | 112.7 ± 34.2 |
| LV Mass Index (ASE), g/m^2^ | 77.1±17.7 | 80.2±19.5 | | 84.2±22.1 | | 88.6±24.7 | | 92.9±27.7 | | 96.4±29.7 | | 99.5±30.2 | 103.0±61.7 | 110.1±33.6 | 123.1±36.5 |
| Normal diastolic function | 6269 (77.8%) | 6491 (77.2%) | | 5906 (72.1%) | | 5471 (66.7%) | | 5049 (62.0%) | | 4562 (59.1%) | | 4142 (55.9%) | 4038 (53.8%) | 3744 (46.5%) | 2579 (26.3%) |
| Abnormal diastolic function | 18 (0.2%) | 47 (0.6%) | | 119 (1.5%) | | 242 (2.9%) | | 457 (5.6%) | | 626 (8.1%) | | 693 (9.4%) | 770 (10.3%) | 1048 (13.0%) | 1782 (18.2%) |
| Normal filling pressures % | 3450 (42.8%) | 2990 (35.6%) | | 2219 (27.1%) | | 1638 (20.0%) | | 1027 (12.6%) | | 662 (8.6%) | | 411 (5.5%) | 229 (3.0%) | 99 (1.2%) | 11 (0.1%) |
| Indetermined filling pressure, % | 4295 (53.3%) | 4697 (55.9%) | | 4716 (57.6%) | | 4594 (56.0%) | | 4163 (51.1%) | | 3495 (45.3%) | | 2993 (40.4%) | 2674 (35.6%) | 2577 (32.0%) | 2736 (27.9%) |
| Elevated filling pressure, % | 80 (1.0%) | 301 (3.6%) | | 673 (8.2%) | | 1075 (13.1%) | | 1481 (18.2%) | | 1581 (20.5%) | | 1614 (21.8%) | 1776 (23.6%) | 2129 (26.4%) | 2645 (27.0%) |
| **AI-LVD Profile** | | | | | | | | | | | | | | | |
| Key echo variables reported, 0-8 | 5.26 ± 1.65 | 5.22 ± 1.73 | | 5.11 ± 1.81 | | 4.93 ± 1.95 | | 4.62 ± 2.17 | | 4.26 ± 2.34 | | 3.85 ± 2.47 | 3.55 ± 2.52 | 3.56 ± 2.43 | 4.15 ± 2.04 |
| AI-LVD Probability Score, 0-1.00 | 0.419 ± 0.001 | 0.423 ± 0.001 | | 0.426 ± 0.001 | | 0.430 ± 0.002 | | 0.437 ± 0.002 | | 0.445 ± 0.003 | | 0.457 ± 0.004 | 0.474 ± 0.005 | 0.495 ± 0.007 | 0.535 ± 0.018 |
| **Follow up** | | | | | | | | | | | | | | | |
| All-cause mortality, % | 452 (5.6%) | 549 (6.5%) | 791 (9.7%) | | 1132 (13.8%) | | 1511 (18.5%) | | 1801 (23.3%) | | 2224 (30.0%) | | 2877 (38.3%) | 3833 (47.6%) | 6136 (62.5%) |
| Actual 1-year mortality, % | 121 (1.5%) | 137 (1.7%) | 205 (2.6%) | | 322 (4.1%) | | 396 (5.0%) | | 541 (7.2%) | | 742 (10.3%) | | 979 (13.4%) | 1432 (18.2%) | 2753 (28.9%) |
| Actual 5-year, mortality, % | 323 (6.6%) | 379 (7.7%) | 575 (12.5%) | | 793 (17.4%) | | 1045 (24.1%) | | 1308 (32.6%) | | 1660 (42.9%) | | 2244 (57.5%) | 3117 (70.3%) | 5304 (89.4%) |

**Legend**: Number of cases with a documented – body mass index (n=59,332), systolic (n=11,925)/diastolic (n=11,876) BP, TR peak velocity (n=44,772), RA volume index (n=22,080), LAVi (n=28,386), LVEF (n=67,521), stroke volume index (n=22,234), E/e’ ratio (n=36,842), E wave velocity (n=65,203), and LV mass index (n=47,791). Full 1- and 5-year mortality calculable in 71,600 and 34,515 cases, respectively. Key echo variables are based on the 8 clinical variables identified as the most important in deriving the AI-LVD.

**Supplemental Material 1: Feature Importance and Panel of Experts**

Detailed explainability experimentation was performed, informed by consultation with a panel of experts that confirmed the AI-LVD’s capacity to detect increasing levels of LV dysfunction. Three distinct processes took place. First, a **feature importance** analysis was undertaken by the engineering and data science team to identify the features that had the most impact on model performance when removed. Each feature was ordered and placed in a grid containing detailed statistical analysis of the impact on the imputation model and the final output of the model. Importantly, this process involved only echocardiographic measurement data and no outcomes were available (specifically, no mortality data). Second, a **panel of expert echocardiographers** then reviewed each measurement and **identified closely associated variables** (for example, the association between septal e’ velocity and the E:e’ ratio, the parasternal long axis systolic dimension and the Teichholz systolic volume, or left atrial area and left atrial volume) which could be concatenated into a single, clinically relevant variable that would be routinely measured in clinical echocardiography. Third, the panel of experts then chose the **variables with the highest feature importance that were clinically relevant** and would be expected to be performed routinely in a standard echocardiogram (for example, right atrial volume had high feature importance, but was not chosen by the panel since it may not be routinely measured).

**Supplemental Figure 1: Test set AI-LVD model characteristics. Panel A** shows 1-D partial dependence plots for important variables, demonstrating the change in average output probability score over the range of each variable. Black horizontal lines indicate the global mean output probability score for the test set. **Panel B** shows 2-D contour maps of the partial dependence across pairs of important variables, demonstrating the effect on the average output probability score of varying both simultaneously. These dependence characteristics along with heart failure and diastolic function guidelines, were considered by a panel of expert echocardiographers, and only the most influential 8 variables were chosen to be “minimum required” data for a probability output to be displayed.

Panel A


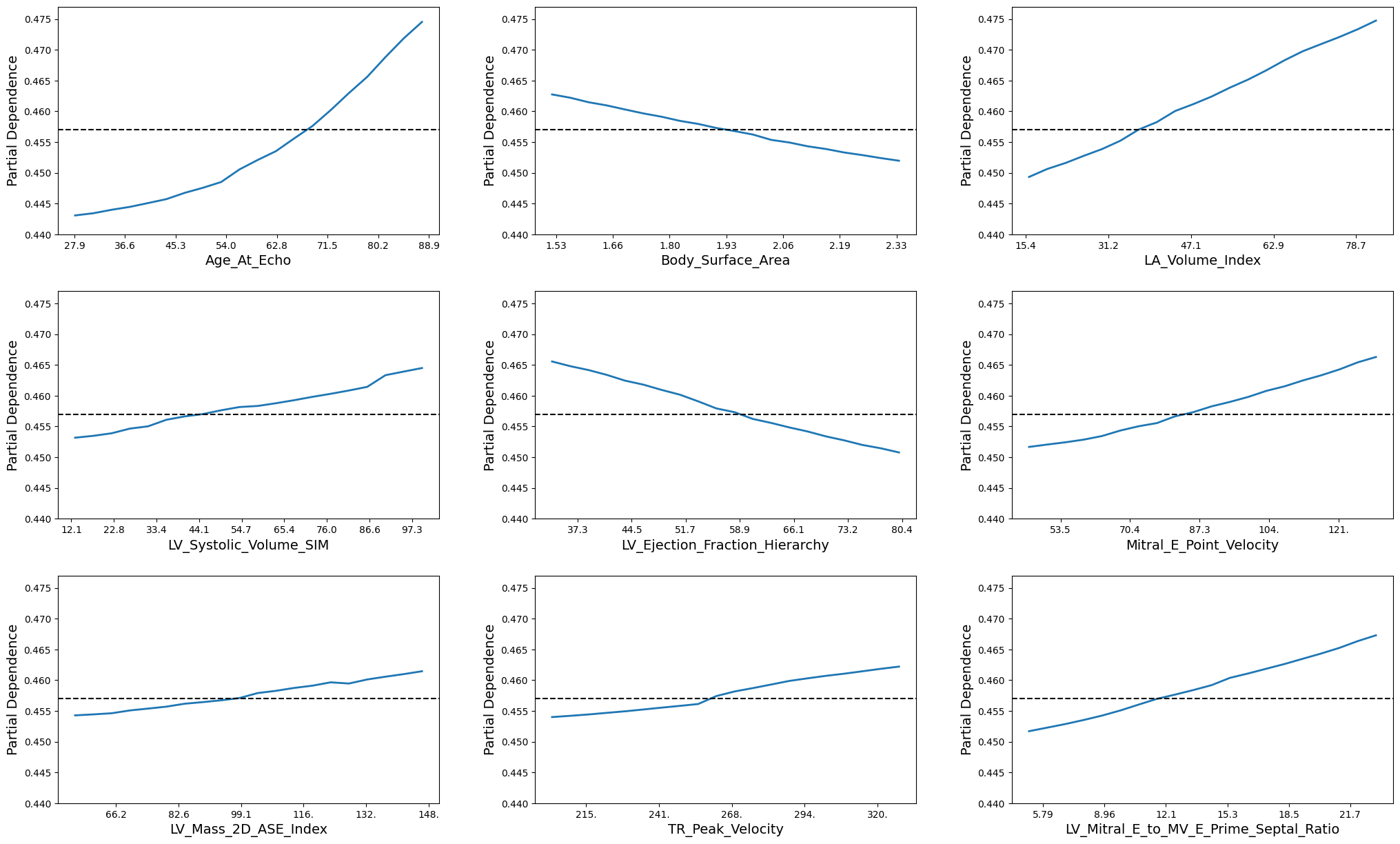


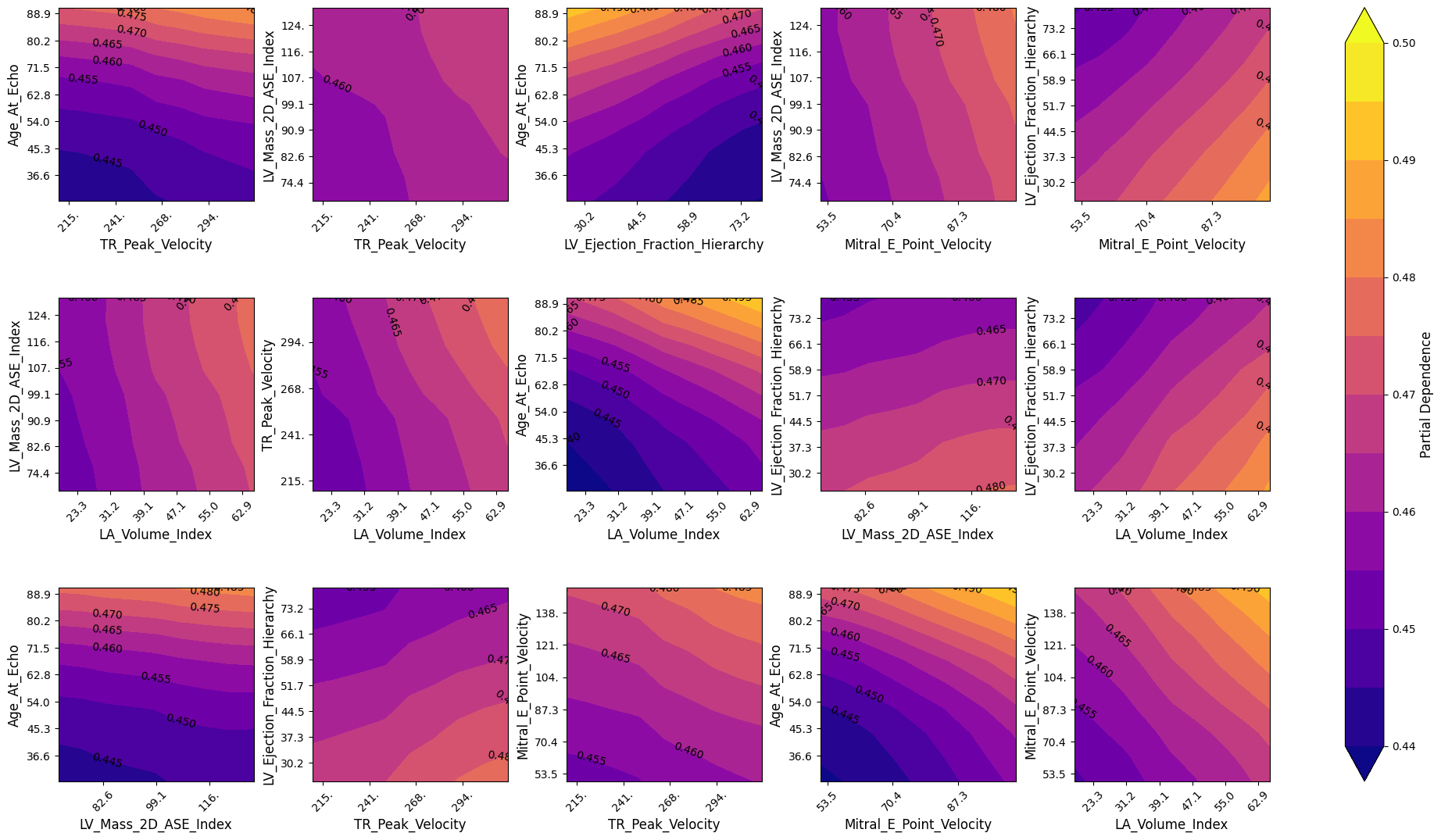
**Supplemental Figure 2**: The distribution of the AI-LVD probability output for the test group (men and women combined) with increasing numbers of key variables included (starting with zero inputs in **Panel A**, top left, increasing to three inputs, **Panel D**, bottom right). The positive skew shows a distribution toward the lower probability output for most echos when exposed to a higher number of variables. The model
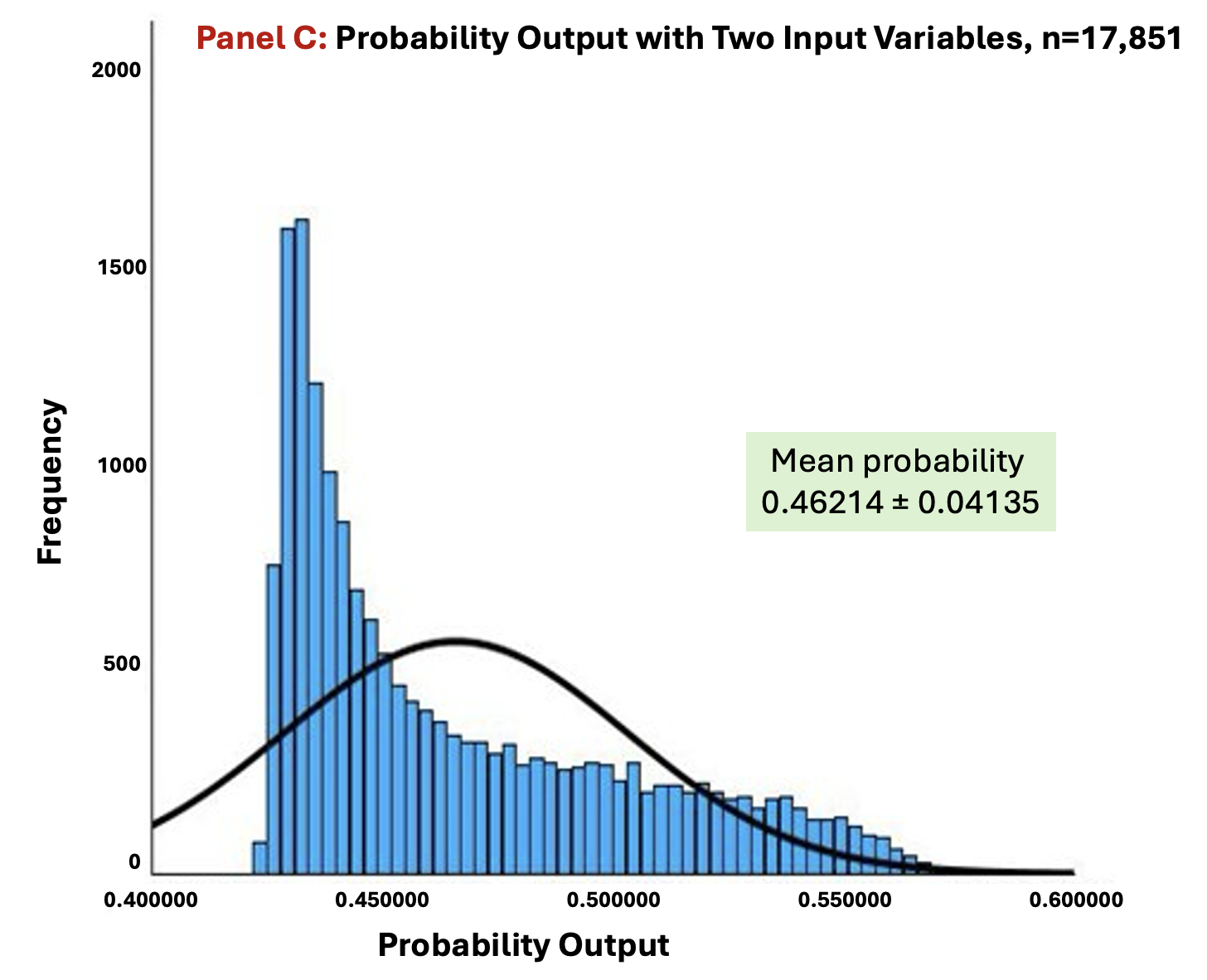

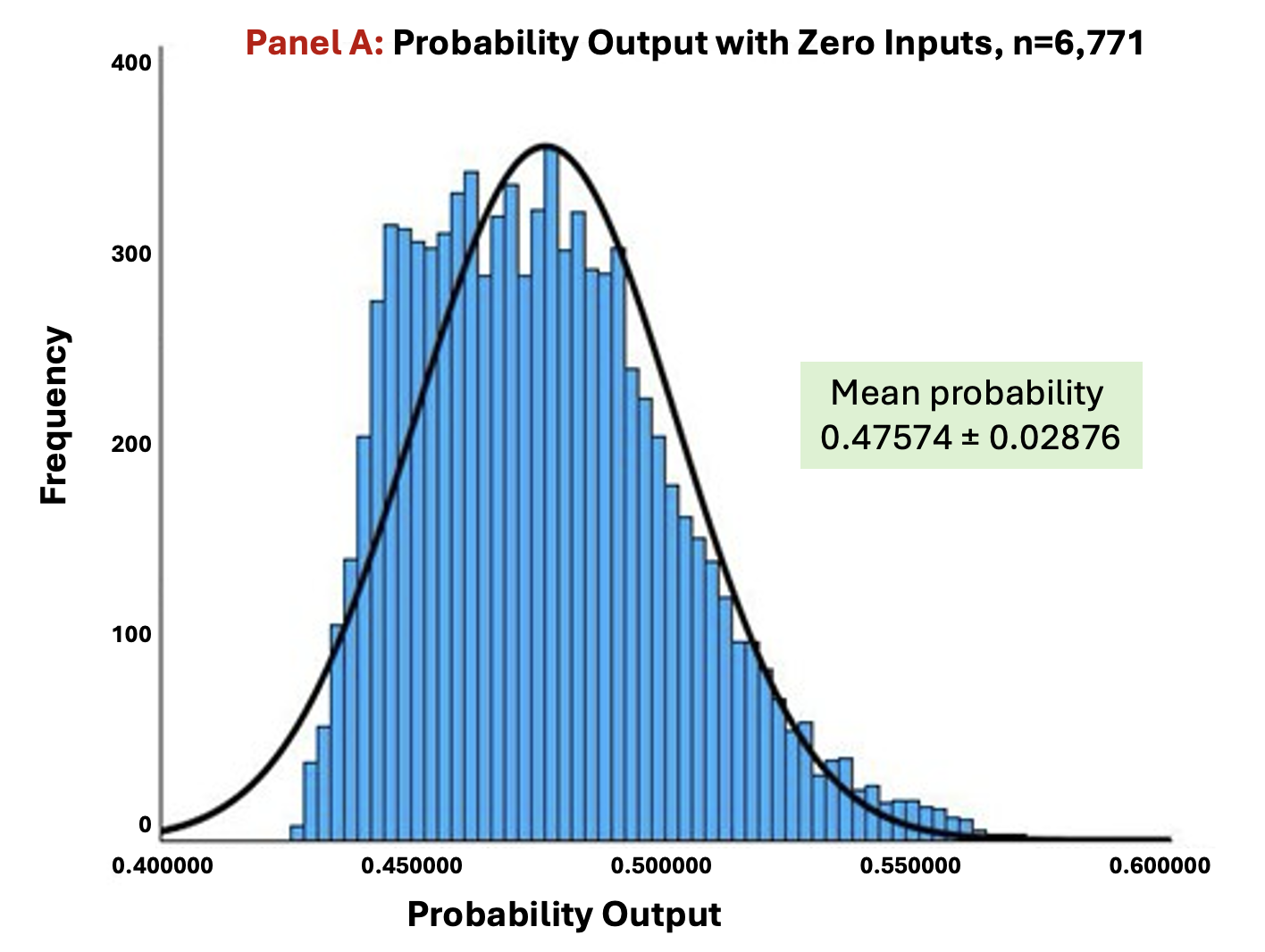

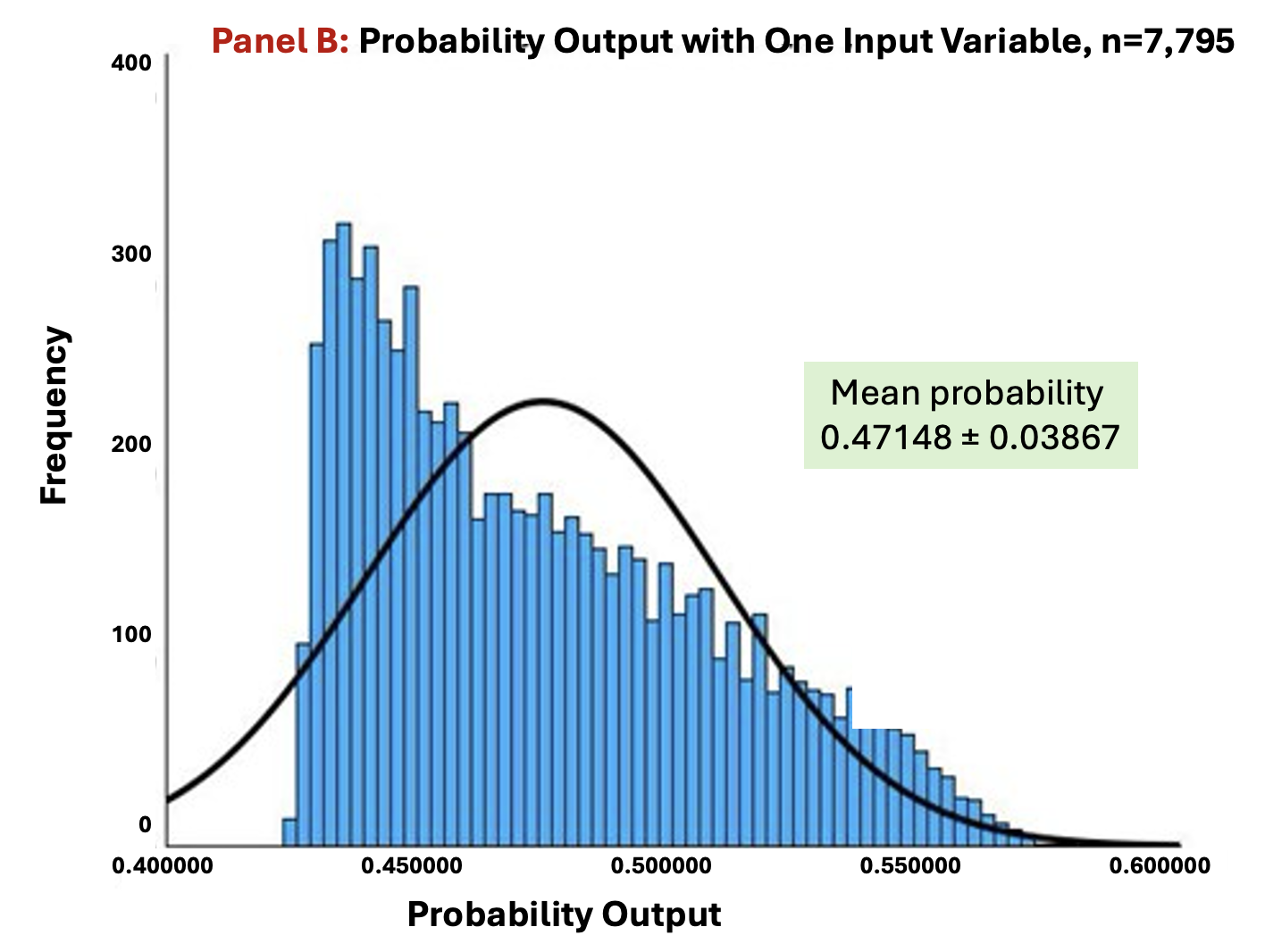
performs optimally when exposed to *all measured* echocardiographic variables.

Panel B


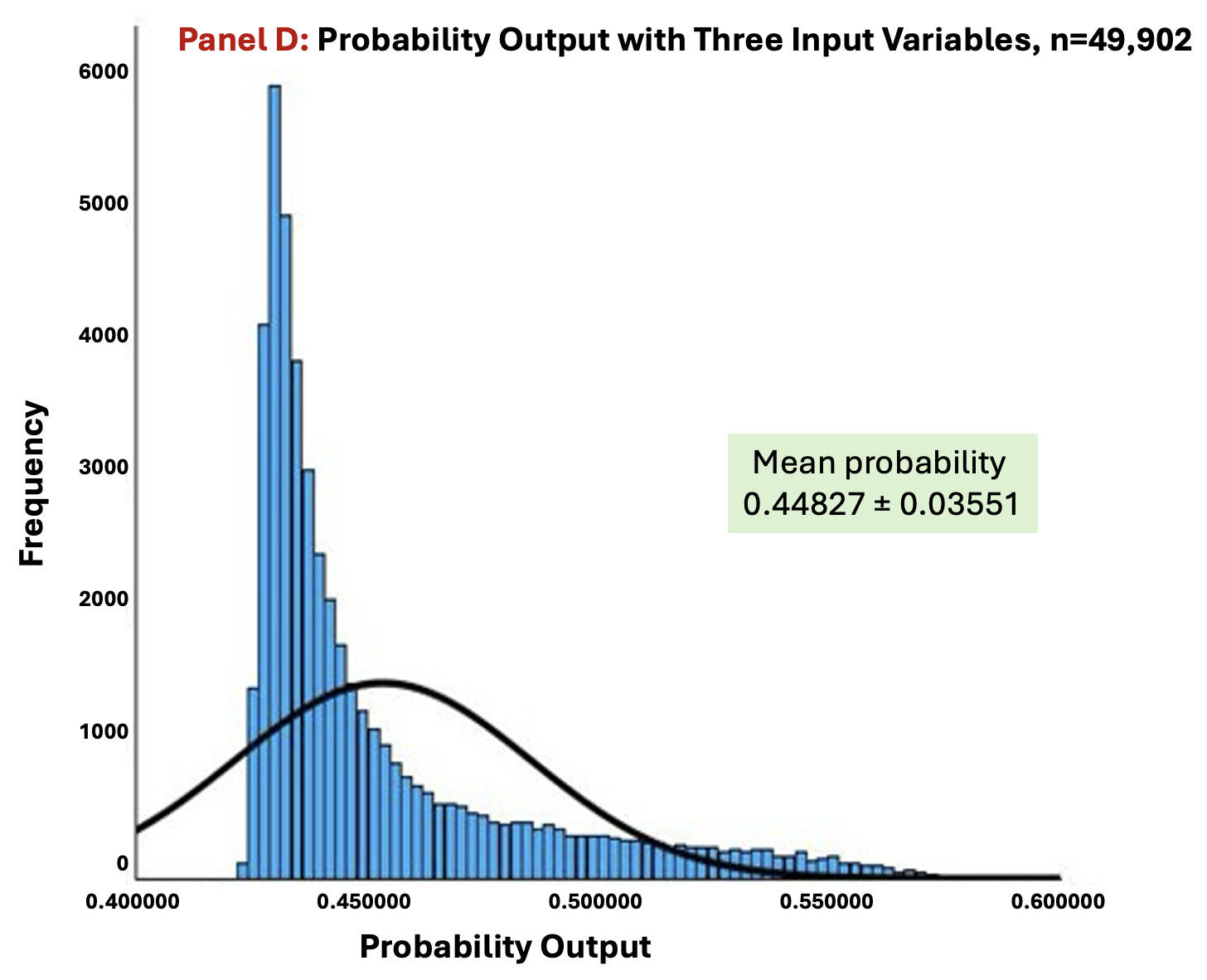


**Supplemental Figure 3:** Sex-specific distribution of key echo variables to determine LVD. LVEF = left ventricular ejection fraction. Mitral E velocity = Mitral inflow maximum E wave velocity. BSA = Body Surface Area, calculated using the Mostellar equation. LVMi = Left Ventricular Mass Indexed for body surface area, calculated using the basal 2D American Society of Echocardiography formula. TRV = Peak tricuspid regurgitation velocity. E:e’ = ratio of the mitral inflow E wave velocity and the septal mitral annular early relaxation (e’) velocity. LAVi = Indexed left atrial volume using the apical biplane method of disks. LVSV = left ventricular systolic volume, calculated using the apical biplane method of disks.

**Supplemental Figure 4**: Pattern of increasing mortality (men and women combined) for increasing AI-LVD deciles in reduced LVEF (rEF, **Panel A**), mildly reduced LVEF (mrEF, **Panel B**), preserved LVEF (pEF, **Panel C**), and “indeterminate” cases where LVEF was not reported or calculable (Indeterminate, **Panel D**).

|  |  |
| --- | --- |
|  |  |

**Legend:** This graphs plot the Cox-proportional hazards for long-term all-cause mortality adjusted for age, year of (last) echo (reference group for comparison – 2000-04), sex (reference group for comparison – women), the number of key echo variables reported/available to the AI-LVD (0-8), and the decile distribution of AI-LVD scores in men (reference group for comparison – lowest decile group being an output probability output of <0.421).

**Supplemental Figure 5**: **Complete partial dependence characteristics for the AI-LVD model. Panel A** shows 1-D partial dependence plots, demonstrating the change in average output probability score over the range of each variable. Black horizontal lines indicate the global mean output probability score for the test set. **Panel B** shows 2-D contour maps of the partial dependence across pairs of variables, demonstrating the effect on the average output probability score of varying both simultaneously.

Panel A


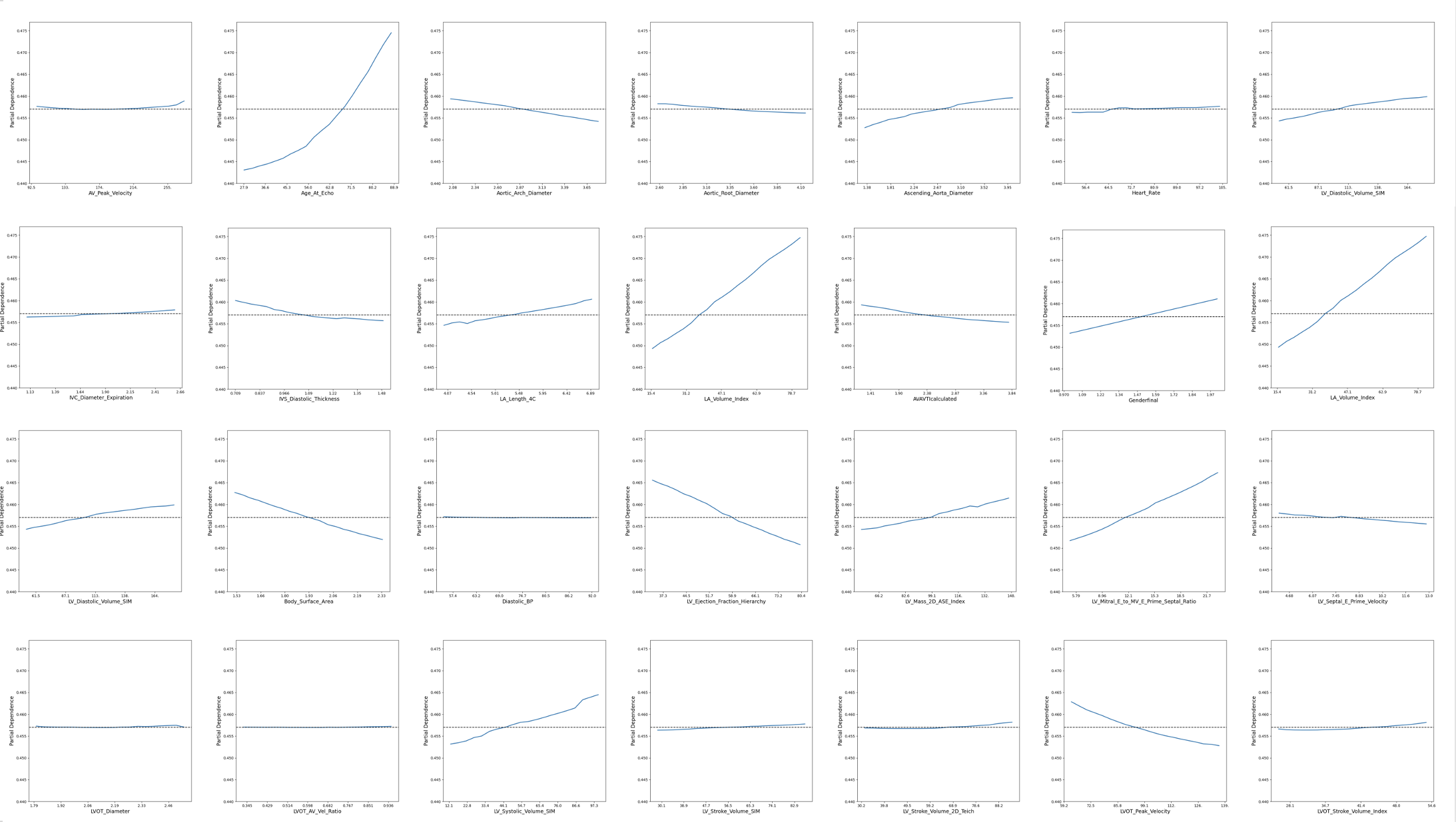


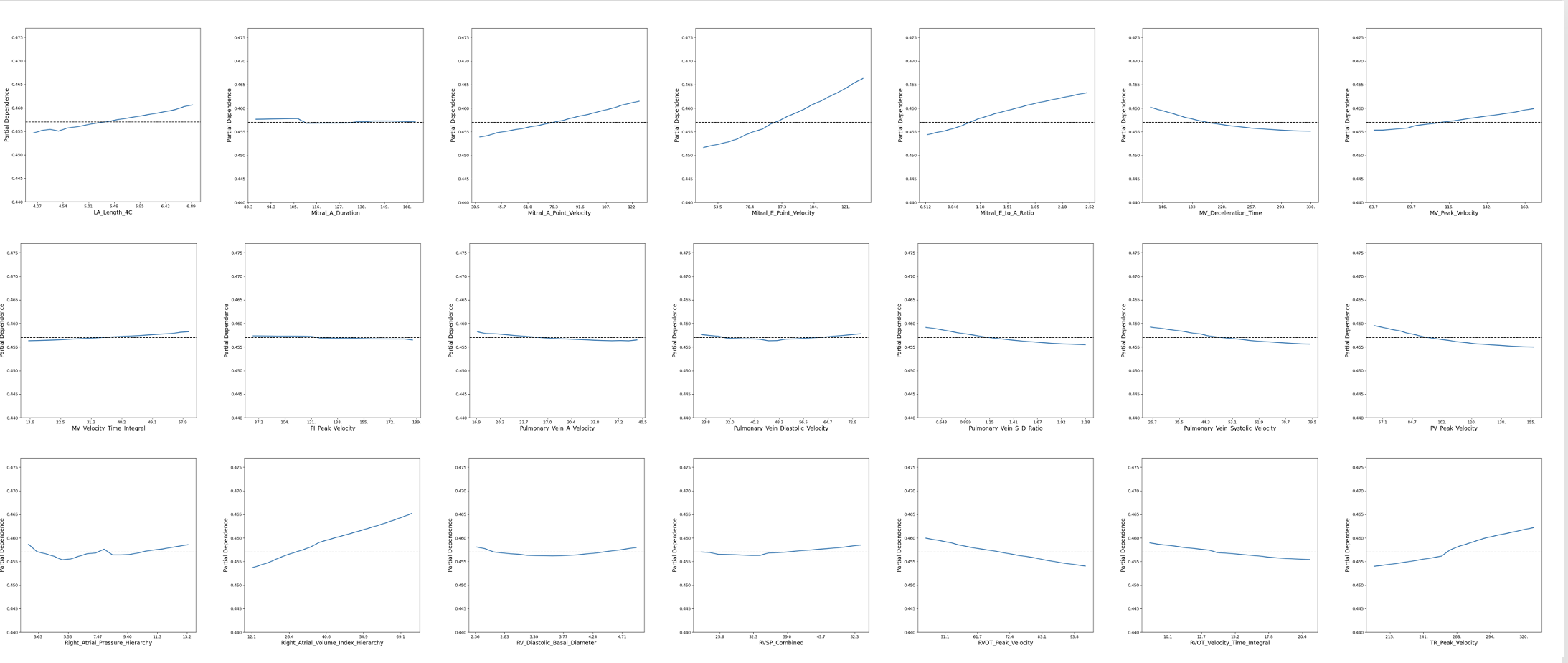


Panel B


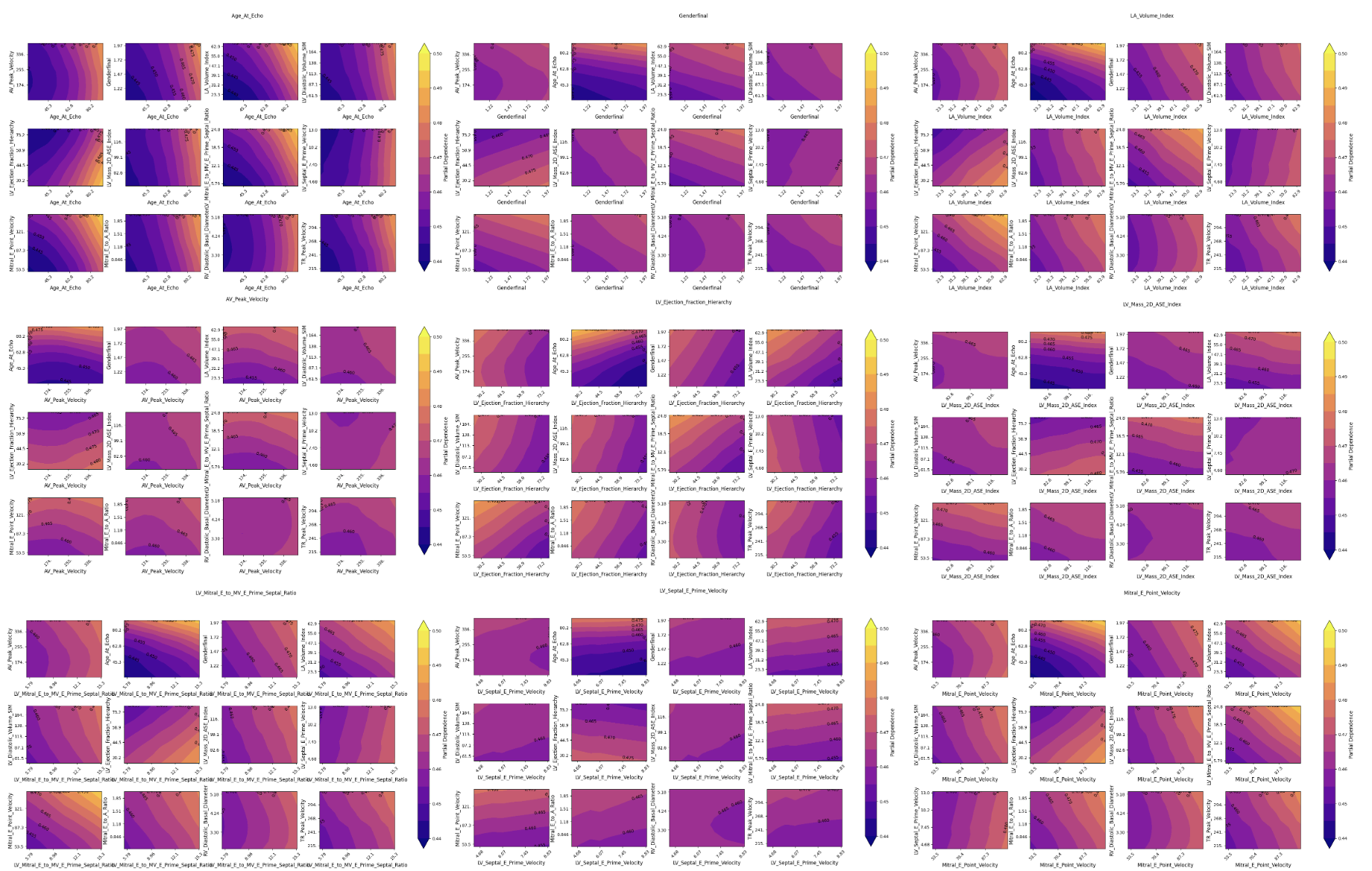


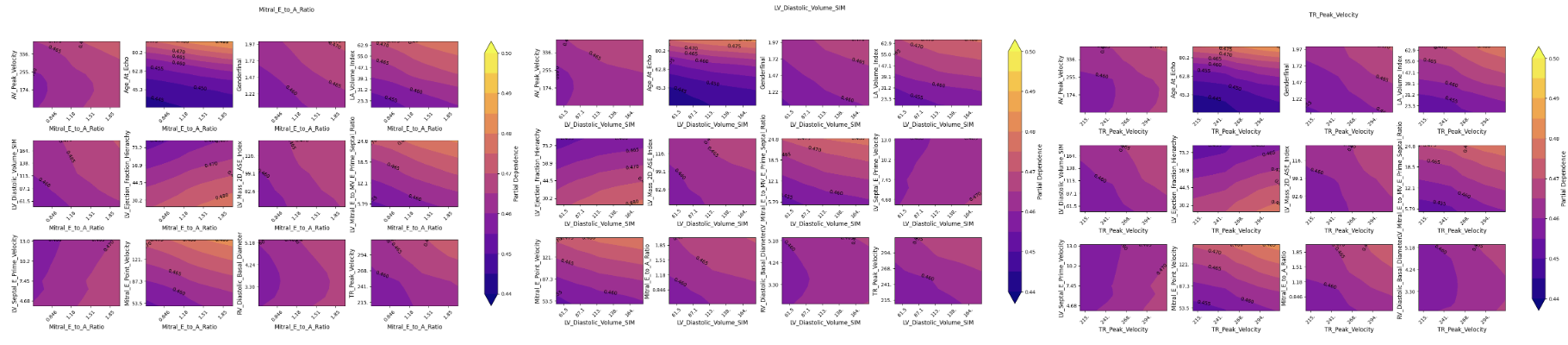


**Supplemental Table 2: The PRIME checklist for reporting echocardiographic AI studies.**

| **Section** | **Checklist item** | **Page #** |
| --- | --- | --- |
| **1** | **Designing the Study Plan** |  |
| 1.1 | Describe the need for the application of machine learning to the dataset | 5 |
| 1.2 | Describe the objectives of the machine learning analysis | 5 |
| 1.3 | Define the study plan | 4 |
| 1.4 | Describe the summary statistics of baseline data | 8 |
| 1.5 | Describe the overall steps of the machine learning workflow | 5-6 |
| **2** | **Data Standardization, Feature Engineering, and Learning** |  |
| 2.1 | Describe how the data were processed in order to make it clean, uniform, and  consistent | 4 |
| 2.2 | Describe whether variables were normalized and if so, how this was done | N/A |
| 2.3 | Provide details on the fraction of missing values (if any) and imputation methods | 6 |
| 2.4 | Describe any feature selection processes applied | 5 |
| 2.5 | Identify and describe the process to handle outliers, if any | 5 |
| 2.6 | Describe whether class imbalance existed and which method was applied to deal with it | 5 |
| **3** | **Selection of Machine Learning Models** |  |
| 3.1 | Explicitly define the goal of the analysis e.g., regression, classification, clustering | 5 |
| 3.2 | Identify the proper learning method used (e.g., supervised, reinforcement learning etc.) to address the problem | 5 |
| 3.3 | Provide explicit details on the use of simpler, complex, or ensemble models | 5 |
| 3.4 | Provide the comparison of complex models against simpler models if possible |  |
| 3.5 | Define ensemble methods, if used | N/A |
| 3.6 | Provide details on whether the model is interpretable |  |
| **4** | **Model Assessment** |  |
| 4.1 | Provide a clear description of data used for training, validation, and testing | 5 |
| 4. 2 | Describe how the model parameters were optimized (e.g., optimization technique, number of model parameters etc.) | 5 |
| **5** | **Model Evaluation** |  |
| 5.1 | Provide the metric(s) used to evaluate the performance of the model | 5,6 |
| 5.2 | Define the prevalence of disease and the choice of the scoring rule used | 5,6 |
| 5.3 | Report any methods used to balance the numbers of subjects in each class | N/A |
| 5.4 | Discuss the risk associated to misclassification | 11-12 |
| **6** | **Best Practices for Model Replicability** |  |
| 6.1 | Consider sharing code or scripts on a public repository with appropriate copyright protection steps for further development and non-commercial use | 6 |
| 6.2 | Release a data dictionary with appropriate explanation of the variables | Supp data |
| 6.3 | Document the version of all software and external libraries used |  |
| **7.** | **Reporting Limitations, Biases and Alternatives** |  |
| 7.1 | Identify and report the relevant model assumptions and findings | 13 |
| 7.2 | If well performing models were tested on a hold-out validation dataset, detail the  data of that validation set with the same rigor as that of training dataset (see section 2 above) | N/A |
